# Recurrences in stage II rectal carcinoma after curative resection alone: from the viewpoint of angiogenesis

**DOI:** 10.1186/s12957-016-0877-6

**Published:** 2016-04-22

**Authors:** Željko Martinović, Dražen Kovač, Cvita Martinović

**Affiliations:** Department of Surgery, Croatian Hospital “Dr. Fra Mato Nikolić”, 72 276 Nova Bila, Bosnia and Herzegovina; Department of Pathology, School of Medicine, University of Rijeka, 51 000 Rijeka, Croatia; Department of Internal Medicine, Croatian Hospital “Dr. Fra Mato Nikolić”, 72 276 Nova Bila, Bosnia and Herzegovina

**Keywords:** Rectal cancer, Angiogenesis, Microvessel density, Endoglin, Recurrence

## Abstract

**Background:**

Angiogenesis plays a pivotal role in malignant tumor progression. The count of blood microvessels of the tumor has been recognized as an indicator of malignant potential of the tumors and provides the ability to predict tumors recurrence. The role endoglin in the Dukes B rectal cancer is still unexplored. The aims of this study were to examine immunohistochemical expression of endoglin in resected rectal cancer and investigate the relationship of tumor recurrence and other clinicopathological variables to the endoglin-assessed microvessel density of the tumor tissue and distal resection margins.

**Methods:**

The study included 95 primary rectal adenocarcinomas, corresponding to 95 distal and 95 proximal resection margin specimens from surgical resection samples. Tumor specimens were paraffin embedded, and immunohistochemical staining for the CD105 endothelial antigen was performed to count CD105-MVD. For exact measurement of the CD105-MVD used, a computer-integrated system Alphelys Spot Browser 2 was used.

**Results:**

The MVD was significantly higher in the tumor samples compared with the distal resection margins (*p* < 0.0001) and the proximal resection margins (*p* < 0.0001). There was no significant difference in the MVD between distal and proximal resection margins (*p* = 0.147). The type of surgical resection was a significant factor for determining the recurrence of tumors (*p* = 0.0104). There was no significant effect of patients’ age, gender, tumor location, grade of differentiation, histological tumor type, and the size and depth of tumor invasion on the recurrence of the tumor. The recurrence rate was significantly higher in the low CD105-MVD group of patients than in the high CD105-MVD group of patients (log rank test, *p* = 0.0406). Result of the multivariate analysis showed that the type of surgery (*p* = 0.0086), MVD tumors (*p* = 0.0385), and MVD of proximal resection margin (*p* = 0.0218) were the independent prognostic factors for the recurrent tumors.

**Conclusions:**

CD105-assessed MVD could help to identify patients with more aggressive disease and increased risk of developing tumor recurrence after surgical treatment in stage II rectal cancer (RC).

## Background

Radical surgical treatment of stage II rectal cancer (RC) is surgical challenge associated with high risk of recurrence of the tumor [1]. After total mesorectal excision surgery with intent, despite the absence of nodal disease, 25 % of these patients will relapse within 5 years [2, 3]. Recurrence of the tumor is an adverse prognostic indicator with a poor overall survival prognosis [4]. The risk of relapse may be estimated by assessing the clinical and histopathological features of the cancer [5].

Tumor growth and its spread to adjacent tissue depend on its ability to stimulate angiogenesis. Angiogenesis consists of formation of new blood vessels from pre-existing vasculature [6]. The studies have shown that the angiogenic potential of a tumor may be inferred from its vascularity measured in histological section [7]. The count of blood microvessels of the tumor, as shown in microvessel density (MVD), has been recognized as an indicator of malignant potential of the tumors and provides the ability to predict tumor recurrence and survival. Endoglin (CD105) has been suggested to be the most suitable marker available to quantify tumor angiogenesis [8].

Our study aimed to examine immunohistochemical expression of CD105 in stage II RC and to investigate a correlation between CD105-assessed MVD and clinicopathological variables and to analyze prognostic value of MVD on the tumor recurrence.

## Methods

### Patients and specimens

We studied 95 cases of primary rectal adenocarcinomas in stage II (T3–T4, N0, M0) treated by complete surgical resection (R0) in a 5-year period at Clinic for Surgery, Clinical Hospital Center Rijeka, Croatia, from January 2002 to December 2006. The study included 95 primary rectal adenocarcinomas, 95 surgical distal resection margin specimens, and 95 surgical proximal resection margin specimens from surgical resection samples. The distal resection margin (DRM) and proximal resection margin (PRM) corresponding to the primary tumor from the same patients was taken from the margin of near and distant surgical resection. Tissue samples included in this study were retrieved from the archives of the Institute of Pathology School of Medicine of Rijeka, Croatia. The exclusion criteria were a synchronous tumor or tumors in another localization in anamnesis, emergency surgery, preoperative radiotherapy or chemotherapy, perforation of bowel, and incomplete clinical data. The study was approved by the University of Rijeka Ethics Committee and patients signed informed consent.

All of the patients underwent radical low anterior resection (LAR) or abdominoperineal resection (APR). All patients had confirmed rectal adenocarcinomas by histopathology and were staged according to the 7th edition of the American Joint Committee on Cancer Staging Manual [9]. The histological grading was classified according to the World Health Organization (WHO) classification [10]. The mean duration of follow-up was 54.7 ± 23.1 months (median duration, 60.0 months) after the operation for RC. Recurrence data and cause of death of those who died during follow-up period were obtained from the Croatian Cancer Registry. Patient and tumor characteristics are presented in Table 1.

**Table 1:** Clinical and pathologic characteristics of the rectal cancer samples

Characteristics	Number of patients
Total number	95
Age, median 69 years	
≤69	49
>69	46
Gender	
Male	61
Female	34
Type of surgery	
Low anterior resection	76
Abdominoperineal resection	19
Tumor location	
Upper rectum	23
Middle rectum	52
Low rectum	20
Grade of differentiation	
G1	55
G2	34
G3	6
Histologic type	
Adenocarcinoma	82
Adenocarcinoma with mucinous features	13
Depth of tumor invasion	
T3	37
T4a	42
T4b	16
Tumor size	
≤4 cm	64
>4 cm	31
Tumor recurrence	
Yes	16
No	79

### Immunohistochemistry

Immunohistochemical analysis was performed on formalin-fixed paraffin-embedded section. All tissue samples from RC, DRM, and PRM were fixed in 10 % buffered formalin and embedded in paraffin. We prepared 4-μm-thick serial section which were deparaffinized in xylene, rehydrated in graded ethanol, and washed with phosphate-buffered saline. Endogenous peroxidase was inhibited with 3 % hydrogen peroxide. Tissue sections were incubated for 30 min with the anti-CD105 primary monoclonal antibody (mouse anti-human, clone SN6h, Dako Corporation, Denmark) at a 1:10 dilution. Primary antibody binding site was visualized using a secondary antibody detection kit (Envision + kit; Dako, Denmark).

The staining was visualized with diaminobenzidine (DAB). Tissue sections were counterstained with hematoxylin. Brown staining for CD105 was considered positive. Distant normal mucosa free of tumor were used as positive controls, and the primary antibody was replaced with phosphate-buffered saline solution for negative controls.

### Evaluation of staining and of MVD by computerized image analysis

All slides stained with anti-CD105 were viewed and analyzed with Alphelys Spot Browser 2 integrated system, using a software controlled (Alphelys Spot Browser 2.4.4., France) stage positioning Nikon Eclipse 50i microscope mounted 1360 × 1024 resolution Microvision CFW-1310C digital camera. The slides were scanned at ×20 magnification to identify “hot spots” (areas with the highest microvessel concentration) for the slides and then ×200 magnification to create images for quantification scoring positive cells and MVD. Positive cells were counted in the tumor, DRM, and PRM and presented as percentage of positive cells and MVD as number of microvessels in the histological field according to Weidner et al. [11]. The regions with the most intensive vascularization (hot spots) were defined by scanning the entire tumor section at low magnification with a selection of four fields. The areas of this histological field was 0.612 mm^2^. Hot spots were identified by two independent observers at ×20 magnification.

### Statistical analysis

Statistical analysis was performed using MedCalc version 14.8.1 (MedCalc Software bvba, Mariakerke, Belgium). Descriptive statistics and 95 % confidence intervals were calculated to describe data. The distribution of data was tested for normality using the Smirnov-Kolmogorov test. The Mann-Whitney *U* test and Kruskal-Wallis tests were used to compare MVD among the clinicopathological variables. Spearman’s rho correlation was used to test the correlation between the immunohistochemical findings and tumor recurrence. The receiver operating characteristic (ROC) curve approach was used to determine best-fitting cut-off for the MVD in terms of the tumor recurrence analysis [12]. Tumor recurrence analysis was estimated by the Kaplan-Meier method and compared by the log rank test. Prognostic factors of tumor recurrence were identified by the use of the Cox proportional hazard regression. Differences at *p* < 0.05 were considered significant.

## Results

### Patient sample classification

We assessed paraffin-embedded specimens from tumors from the 95 patients resected for RC. Clinicopathological characteristics of patients are summarized in Table 1. The median age at diagnosis was 69 years (range 15 to 85 years), 49 patients (51.6 %) were ≤69 years of age, and 46 patients (48.4 %) were >69 years old. Sixty-one (64.2 %) were males and 34 (35.8 %) were females. In 23 patients (24.2 %), the tumor was located in the upper rectum, in 52 (54.7 %), in the middle rectum and in 20 (21.1 %), in the low rectum. According to the grade of differentiation, 55 patients (57.9 %) were G1 (well differentiated), 34 (35.8 %) G2 (moderately differentiated), and 6 (6.3 %) G3 (poorly differentiated). According to the depth of tumor invasion, 37 patients (38.9 %) were T3, 42 (44.2 %) T4a, and 16 (16.9 %) T4b. Eighty-two (86.3 %) tumors were classified as adenocarcinomas and 13 (13.7 %) as adenocarcinomas with mucinous features. Median tumor size was 3.8 cm (range, 1.3 to 12.0 cm). The median patients follow-up was 60 months (range, 1.0 to 109.0 months). Of the 95 patients, 16 patients developed recurrence of the tumor (recurrence rate, 16.8 %) and 29 died of RC (overall survival rate, 30.5 %) in the 5-year follow-up period.

### Microvessel density

**Fig. 1:**
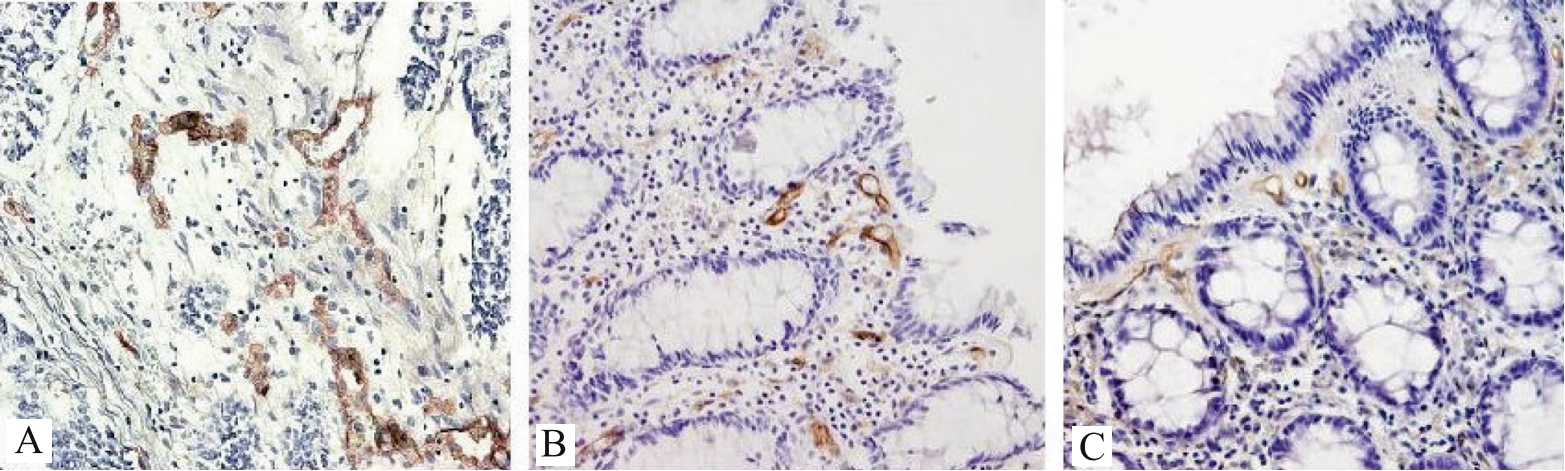
Immunohistochemical staining for the CD105 endothelial antigen in the tumor samples (**a**), distal resection margins (**b**), and the proximal resection margins (**c**). Magnification ×200

**Fig. 2:**
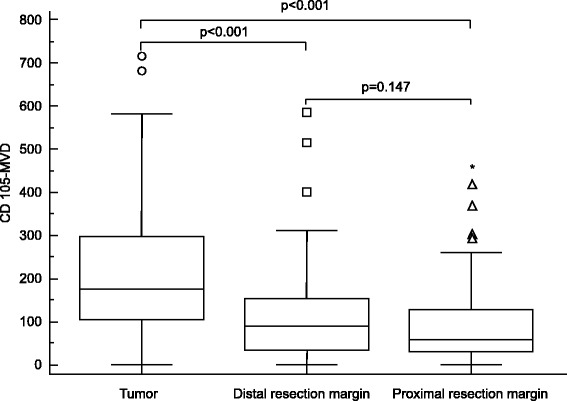
Box-and-whisker plots of CD105-MVD in tumors, distal resection margins, and proximal resection margins. The CD105-MVD level was significantly higher in the tumor compared with the distal resection margin (*p* < 0.001) and the proximal resection margin (*p* < 0.001). In these box plots, median values are represented by *lines within the boxes*, *whiskers* represent the interquartile range, and outliers are represented as individual data points

**Fig. 3:**
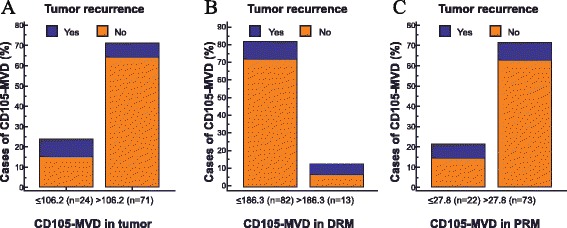
Correlation analysis between tumor recurrence and CD105-MVD in tumor, distal and proximal resection margin. **a** Recurrence and CD105-MVD in tumor (*p* = 0.0037). **b** Recurrence and CD105-MVD in DRM (*p* = 0.0076). **c** Recurrence and CD105-MVD in PRM (*p* = 0.0490)

MVD were analyzed in tumors, distal resection margins, and proximal resection margins. Examples of CD105 expression in the tumor samples, distal resection margins, and the proximal resection margins are shown in Fig. 1. Median CD105-assessed MVD in tumors was 174.47 vessels/mm^2^ (95 % CI 151.00–205.29), distal resection margins 89.86 vessels/mm^2^ (95 % CI 55.46–103.80), and the proximal resection margins 58.82 vessels/mm^2^ (95 % CI 51.42–82.56). The MVD was significantly higher in the tumor samples compared with distal resection margins (Wilcoxon test, *p* < 0.0001) and the proximal resection margins (Wilcoxon test, *p* < 0.0001) (Fig. 2). There was no significant difference in the MVD between distal and proximal resection margins (Wilcoxon test, *p* = 0.147). We found that CD105-MVD in tumor tissue (rho = −0.321, *p* = 0.0015, 95 % CI −.491 to −1.128) and proximal resection margins (rho = −0.220, *p* = 0.0324, 95 % CI −0.403 to −0.019) correlated inversely with tumor recurrence rate. On the other hand, CD105-MVD in distal resection margins (rho = 0.312, *p* = 0.0021, 95 % CI 0.118 to 0.483) correlated positively with tumor recurrence rate. There was statistically significant correlation between CD105-MVD in tumor (≤106.2 vessels/mm^2^/>106.2 vessels/mm^2^) (Fig. 3a; *p* = 0.0037), CD105-MVD in DRM (≤186.3 vessels/mm^2^/>186.3 vessels/mm^2^ (Fig. 3b; *p* = 0.0076), CD105-MVD in PRM (≤27.8 vessels/mm^2^/>27.8 vessels/mm^2^) (Fig. 3c; *p* = 0.0490), and tumor recurrence as determined by Fisher’s exact test.

### Univariate recurrence analysis

**Fig. 4:**
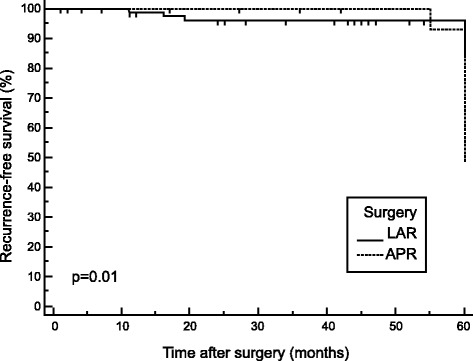
Kaplan-Meier curves for recurrence-free survival according to the type of surgery. *Solid line*, patients with LAR. *Dotted line*, patients with APR

The Kaplan-Meier method and log-rank test were performed. In the univariate analysis (Table 2), the type of surgical resection was a significant factor for determining the recurrence of tumors (log-rank test, *p* = 0.0104) (Fig. 4). There was no significant effect of patients’ age, gender, tumor location, grade of differentiation, histological tumor type, the size and depth of tumor invasion on the recurrence of the tumor.

**Table 2:** Univariate analysis for tumor recurrence

Variable	Hazard ratio	95 % CI	*p* value*
Age (median)			0.716
≤69	1.000		
>69	0.839	0.310–2.268	
Gender			0.142
Male	1.000		
Female	0.426	0.152–1.190	
Type of surgery			0.010
Low anterior resection	1.000		
Abdominoperineal resection	3.140	0.919–10.725	
Tumor location			0.710
Upper rectum	1.000		
Middle rectum	1.267	0.381–4.213	
Low rectum	1.716	0.419–7.026	
Grade of differentiation			0.936
G1	1.000		
G2	1.933	0.416–3.423	
G3	1.119	0.137–9.137	
Histologic type			0.299
Adenocarcinoma	1.000		
Adenocarcinoma with mucinous features	0.381	0.096–1.514	
Depth of tumor invasion			0.925
T3	1.000		
T4a	0.919	0.316–2.673	
T4b	0.745	0.172–3.223	
Tumor size			0.329
≤4 cm	1.000		
>4 cm	0.594	0.214–1.651	

*95 % CI* confidence interval

*log-rank test

**Fig. 5:**
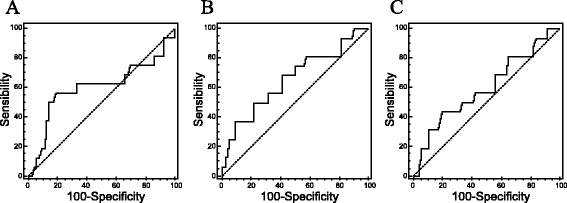
Receiver operating characteristic (ROC) curve analysis for optimal cut-off point of CD105-MVD. **a** Cut-off value of MVD in tumor 106.2/mm^2^. **b** Cut-off value of MVD in DRM 186.3/mm^2^. **c** Cut-off value of MVD in PRM 27.8/mm^2^

The cut-off value for determining high and low MVD with respect to recurrent tumor was performed by the receiver operating characteristic (ROC) curve analysis. The cut-off value for MVD in tumors, distal and proximal resection margins were ≤106.2 microvessel/mm^2^ (sensitivity 56.2 %, specificity 81.0 %), >186.3 microvessel/mm^2^ (sensitivity 37.5 %, specificity 91.1 %), and ≤27.8 (sensitivity 43.7 %, specificity 81.0 %), respectively (Table 3). The cut-off values for MVD in the primary tumors, distal resection margins, and proximal resection margins are shown in Fig. 5.

**Table 3:** Optimal cut-off point of CD105-MVD in tumors, distal resection margins, and proximal resection margins

Variables	AUC	Cut-off value	Sensitivity	Specificity
MVD in tumors	0.601	106.2	56.2	81.0
MVD in DRM	0.656	186.3	37.5	91.1
MVD in PRM	0.602	27.8	43.7	81.0

*AUC* area under the curve

**Fig. 6:**
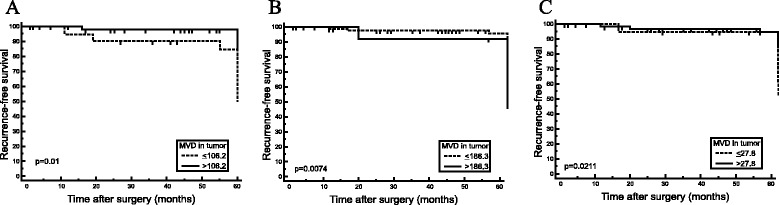
Kaplan-Meier curves for recurrence-free survival according to CD105-MVD. **a** CD105-MVD in tumor (cut-off level of MVD 106.2/mm^2^). **b** CD105-MVD in DRM (cut-off level of MVD 186.3 mm^2^). **c** CD105-MVD in PRM (cut-off level MVD 27.8/mm^2^

In a Kaplan-Meier recurrence of tumors estimate (Table 4), tumor with a low MVD (Fig. 6a, log-rank test, *p* = 0.0008) and tumor with low MVD proximal resection margin (Fig. 6b, log-rank test, *p* = 0.0074) had significantly higher risk of developing recurrence. The risk of developing recurrence was significantly higher in the group of patients with high MVD distal resection margin compared with low MVD distal resection margin (Fig. 6c, log-rank test, *p* = 0.0211).

**Table 4:** The recurrence rates and univariate analysis according to cut-off values for MVD

Variables	Cut-off value	Number of patients	Recurrence rates	*p* value*
MVD in tumors				0.0008
	≤106.2	24	37.5	
	>106.2	71	9.8	
MVD in DRM				0.0074
	≤186.3	82	12.2	
	>186.3	13	46.1	
MVD in PRM				0.0211
	≤27.8	22	31.8	
	>27.8	73	12.3	

*log-rank test

### Multiple Cox regression analysis

The prognostic variables were determined by Cox proportional hazard regression analysis. The variables: age, gender, type of surgical resection, tumor location, grade of differentiation, histological type, depth of tumor invasion, tumor size, length of the surgical distal and proximal margin, MVD tumors, and MVD of surgical distal and proximal margin were entered into the multivariate model to determine their relation with recurrent tumors. “Backward” analysis was performed. Results of the multivariate analysis are presented in Table 5. The result showed that the type surgery (OR = 4.11, *p* = 0.0086), MVD tumors (OR = 0.33, *p* = 0.0385), and MVD of proximal resection margin (OR = 0.27, *p* = 0.0218) were the independent prognostic factors for recurrent tumors. Tumor size and MVD distal resection margins were on the border of significance.

**Table 5:** Cox proportional-hazard regression analysis for tumor recurrence (method “backward”)

Covariate	*p* value	OR	(95 % CI)
Type of surgery (LAR versus APR)	0.0086	4.1103	1.4397–11.7346
Tumor size (≤4 versus >4 cm)	0.0577	0.3356	0.1093–1.0302
MVD in tumors (≤106.2 versus >106.2)	0.0385	0.3330	0.1182–0.9384
MVD in DRM (≤186.3 versus >186.3)	0.0537	2.9359	0.9885–8.7195
MVD in PRM (≤27.8 versus >27.8)	0.0218	0.2778	0.0935–0.8255

Overall model fit *χ* ^2^ = 23.20, *p* = 0.0003

*MVD* microvessel density, *OR* odds ratio, *95 % CI* confidence interval, *DRM* distal resection margin, *PRM* proximal resection margin

## Discussion

Stage II RC is defined by the presence of penetration through the muscularis propria and the absence of metastasis to either regional lymph nodes or distant sites [13]. Rectal cancer surgery is effective for localized disease; however, approximately 25–30 % patients with stage II disease are at high risk for postoperative recurrence, and the clinical outcome of these patients is similar to that of patients with stage III disease [14]. Identifying high-risk patients with stage II RC is important because it may help to identify patients and additional risk for whom surgery alone may not be a curative treatment. During the past two decades, many clinicopathologic studies indicate the importance of tumor angiogenic activity in defining the aggressiveness of tumor behavior [15]. The microvessel density (MVD), which is based on the morphological visualization and quantification of blood vessels, represents a possible prognostic value in colorectal cancer [16]. Over the past decade, numerous studies have investigated the value of angiogenesis markers in CRC. Endoglin is a proliferation-associated antigen on endothelial cells and essential for angiogenesis. It has been reported that expression of the endoglin in tumor endothelium may be a prognostic indicator of the outcome for various human tumors including and colorectal cancer (CRC) [17]. Many angiogenesis markers have been studied but have not been used in conjunction with the angiogenesis in the surgical resection margins.

Microvessel density assessment is the most commonly used technique to quantify intra-tumoral angiogenesis in cancer. In the present study, we assessed MVD with CD105 marker in RC tissue, distal resection margin, and proximal resection margin. In our cohort, endoglin microvessel immunostaining was consistently present in all the cases studied. We showed that the CD105-MVD values significantly increase in RC from the proximal and distal resection margin to the primary tumor (Fig. 2). These results support the role of CD105 as an optimal marker of proliferation of endothelial cells and its potential as prognostic factor [13, 14, 18]. In our study, overall 5-year recurrence rate for all patients included in this study was 16.8 %.

Recurrence rates in our cohort stage II RC patients were analyzed according to age, gender, surgery, tumor location, grade of differentiation, histology, depth of tumor invasion, and tumor size (Table 2). By univariate analysis, only the type of surgical resection was found to be significant prognostic factors for tumor recurrence. Patients treated with a primary APR had a higher rate of tumor recurrence than those who underwent a LAR, which is in accordance with the results of most of the authors [19]. Previous studies have demonstrated an LAR to APR ratio 3:1 or 4:1 which is consistent with our results [19, 20]. However, most studies were too small to adequately evaluate the relationship between the type surgery and tumor recurrence (TR). The key to successful surgery is complete excision of the tumor with sufficient margin of normal tissue. TR may also sometimes occur even in the absence of an involved CRM possibly owing to lymphatic spread from the distal rectum to lymph nodes in the pelvic side wall [21]. In low rectal cancer, total mesorectal excision (TME) surgery may be insufficient to obtain the desired CRM because of lack of mesorectum at the level of the pelvic floor [22]. APR surgery frequently results in perineal and presacral TR [23]. The choice of surgical resection is limited and influenced by tumor staging, tumor location, and intrapelvic tumor invasion at the time of the diagnosis [24].

Patients with stage II RC have a high risk of developing recurrence of the tumor despite multimodality treatment [25]. Although angiogenesis affect the outcome of treatments, the importance of angiogenesis as a prognostic factor is still not clearly enough defined. In the studies, there are considerable differences in microvessel counts in tissue of rectal carcinoma. The quantification of microvessel density was made in the majority of studies with classical Weidner’s method [11]. In our study, tumor microvessel density was obtained by computerized image analysis.

For colorectal cancer, conflicting results have been reported on the prognostic importance of MVD in prediction of tumor aggressive behavior in various subsets of patients. Due to inconsistent methods of analysis of tumor angiogenesis in various studies, it is difficult to compare the values of MVD obtained in our analysis with the results of other authors. In our analysis, we found higher values of MVD (CD105-MVD, 221.0/mm^2^ on average) in RC tissues in comparison with the results in the study of Svagzdys et al. (CD34-MVD, 193.0/mm^2^ on average), possibly due to the larger surface of the analyzed tumor tissue (0.612 versus 0.576/mm^2^) and the use of different endothelial cell markers [26]. In the present study, the microvessel counts are high and confirm that the rectal carcinoma is strongly dependent on angiogenesis.

Furthermore, significantly higher rates of tumor recurrence were found in patients with lower MVD in tumors than in cut-off value obtained by ROC analysis. This is shown by Kaplan-Meier recurrence curve for MVD in RC tissue (Fig. 5). Our results suggest that the lower CD105-MVD is accompanied by higher rate of tumor recurrence, which is not in accordance with the results that an increased CD105-MVD was correlated with recurrence of the disease after radical resection. In their study, Skoufi et al. have found a strong association between increased CD105-MVD and recurrence of CRC [27]. Chen et al. have reported that the RC with higher MVD are more likely to recur or metastasize after radical resection (CD31 immunostaining, average as cut-off) [28]. Other studies demonstrated that high MVD counts determined using CD105 were strongly associated with high risk of metastatic disease (Saad et al., Romani et al., median as cut-off) [29, 30]. According to the results in the study of Uribarrena et al., patients with stage I and II colorectal carcinomas with higher vascularized tumor area had a significant association with a better outcome, but no significant relationship was observed between MVD and tumor recurrence and death [31]. Some studies reveal that MVD determined with CD105 is not correlated with recurrence rate [32, 33].

In the present study, we found a significant inverse correlation between the CD105-MVD in the distal resection margins and the length of DRM that were closest to the RC. This analysis suggested an active reaction of the adjacent mucosa related to the presence of the tumor, but a more passive reaction induced by the factors released from the tumor [34]. Also, our result shows a significant correlation between the CD105-MVD and tumor recurrence: significantly higher rates of tumor recurrence were found in patients with higher CD105-MVD in distal resection margins than cut-off value obtained by ROC analysis. Regarding the pattern of recurrence after surgery alone, we revealed that lower CD105-MVD in tumors and higher CD105-MVD in distal resection margins significantly correlated with tumor recurrence, suggesting that CD105 may be involved in developing tumor recurrence in rectal cancer. However, the results of different studies are linking the lower tumor vascularity with poor outcomes and in various other solid tumors [35]. Only recently acquired knowledge has led to the conclusions that the local tissue microenvironment contributes significantly to tumor progression.

We hypothesize that a crosstalk exists between rectal tumor cells and adjacent mucosa of distal margin. Tumor secretes cytokines and other signaling proteins which induce angiogenesis in the adjacent mucosa of distal margin [34]. TGF-β1, a multifunctional cytokine, has a complex role in angiogenesis. It is expressed in number of cell types, including endothelial cells, depending on the concentration, is both pro angiogenic and antiangiogenic [36]. One molecule that may orchestrate this balance is endoglin [37]. Endoglin is an auxiliary membrane receptor for transforming growth factor beta (TGF-β) that modulates TGF-β signaling [35]. Recently, endoglin has been identified as a key regulator of tumor cells proliferation, migration, and invasion [27, 35]. Craft et al. showed that endoglin expression was lost during prostate cancer cell progression, and that led to increased cell invasion and migration [38]. It has been suggested that endoglin deficiency results in angiogenic adaptation, weakens the endothelial barrier, and increased metastatic spread and may be associated with cancer progression [39].

## Conclusions

In conclusion, this study showed that the CD105-MVD is a useful marker for identifying patients with an aggressive form of stage II RC. CD105-assessed MVD both tumor and adjacent mucosa of distal resection margin could help to identify patients with more aggressive disease and increased risk of developing tumor recurrence after surgical treatment in the group of stage II RC.

## Abbreviations

APR: abdominoperineal resection
CD105: endoglin
CD31: platelet endothelial cell adhesion molecule-1 (PECAM-1)
CD34: hematopoietic progenitor cell antigen CD34
CRC: colorectal cancer
DRM: distal resection margin
LAR: low anterior resection
MVD: microvessel density
PRM: proximal resection margin
RC: rectal cancer
ROC: receiver operating characteristic
TGF β: transforming growth factor beta
TME: total mesorectal excision
TR: tumor recurrence
WHO: World Health Organization
